# Supplementary material for: Modeling glioblastoma heterogeneity as a dynamic network of cell states
Source: Mol Syst Biol. 2021 Sep 16;17(9):e10105. doi: 10.15252/msb.202010105 (PMC8444284; doi:10.15252/msb.202010105)
Supplement: Supplementary file 5 — Source Data for Figure 3 [file MSB-17-e10105-s001.zip › Figure3A_sourcedata/GSEA_3065/nosigdb_state4.GseaPreranked.1622718430994/DOROSZKO_U3180MG_CLUSTER_3.html]

Details for gene set DOROSZKO\_U3180MG\_CLUSTER\_3[GSEA]

|  || Dataset | state4 |
| Phenotype | NoPhenotypeAvailable |
| Upregulated in class | na\_neg |
| GeneSet | DOROSZKO\_U3180MG\_CLUSTER\_3 |
| Enrichment Score (ES) | -0.8001413 |
| Normalized Enrichment Score (NES) | -3.2970755 |
| Nominal p-value | 0.0 |
| FDR q-value | 0.0 |
| FWER p-Value | 0.0 |
Table: GSEA Results Summary

  

Fig 1: Enrichment plot: DOROSZKO\_U3180MG\_CLUSTER\_3      
 Profile of the Running ES Score & Positions of GeneSet Members on the Rank Ordered List

  

| PROBE | GENE SYMBOL | GENE\_TITLE | RANK IN GENE LIST | RANK METRIC SCORE | RUNNING ES | CORE ENRICHMENT || 1 | METRN |  |  | 14 | 0.462 | 0.0028 | No |
| 2 | ETFB |  |  | 315 | 0.172 | -0.0467 | No |
| 3 | TCEAL9 |  |  | 328 | 0.169 | -0.0468 | No |
| 4 | NME4 |  |  | 432 | 0.146 | -0.0628 | No |
| 5 | LRRCC1 |  |  | 606 | 0.125 | -0.0911 | No |
| 6 | IDH2 |  |  | 615 | 0.124 | -0.0911 | No |
| 7 | POLR2I |  |  | 658 | 0.119 | -0.0969 | No |
| 8 | TALDO1 |  |  | 830 | 0.101 | -0.1251 | No |
| 9 | GAMT |  |  | 971 | 0.090 | -0.1480 | No |
| 10 | CSNK2B |  |  | 982 | 0.089 | -0.1488 | No |
| 11 | POLE4 |  |  | 1010 | 0.088 | -0.1524 | No |
| 12 | YWHAQ |  |  | 1251 | 0.073 | -0.1927 | No |
| 13 | INAFM1 |  |  | 1466 | 0.063 | -0.2286 | No |
| 14 | ENOPH1 |  |  | 1638 | 0.057 | -0.2573 | No |
| 15 | AKR7A2 |  |  | 1676 | 0.056 | -0.2630 | No |
| 16 | CCDC167 |  |  | 1711 | 0.055 | -0.2682 | No |
| 17 | MINOS1 |  |  | 2071 | 0.045 | -0.3292 | No |
| 18 | SRR |  |  | 2087 | 0.044 | -0.3313 | No |
| 19 | MZT2B |  |  | 2107 | 0.044 | -0.3340 | No |
| 20 | FKBP3 |  |  | 2130 | 0.043 | -0.3373 | No |
| 21 | TMEM54 |  |  | 2142 | 0.043 | -0.3387 | No |
| 22 | ACADM |  |  | 2224 | 0.041 | -0.3521 | No |
| 23 | SUMO3 |  |  | 2239 | 0.041 | -0.3541 | No |
| 24 | PEG10 |  |  | 2338 | 0.038 | -0.3704 | No |
| 25 | MEA1 |  |  | 2356 | 0.038 | -0.3729 | No |
| 26 | SNX5 |  |  | 2437 | 0.036 | -0.3862 | No |
| 27 | RAC3 |  |  | 2442 | 0.036 | -0.3865 | No |
| 28 | BANF1 |  |  | 2472 | 0.035 | -0.3911 | No |
| 29 | TCTEX1D2 |  |  | 2514 | 0.034 | -0.3977 | No |
| 30 | ATP5MC1 |  |  | 2610 | 0.033 | -0.4136 | No |
| 31 | BCL7C |  |  | 2667 | 0.031 | -0.4229 | No |
| 32 | UQCC2 |  |  | 2852 | 0.027 | -0.4541 | No |
| 33 | MZT2A |  |  | 2994 | 0.025 | -0.4779 | No |
| 34 | PGK1 |  |  | 3012 | 0.025 | -0.4806 | No |
| 35 | PSMB9 |  |  | 3055 | 0.024 | -0.4875 | No |
| 36 | PAFAH1B3 |  |  | 3105 | 0.023 | -0.4956 | No |
| 37 | AP2S1 |  |  | 3110 | 0.023 | -0.4961 | No |
| 38 | MCUR1 |  |  | 3261 | 0.020 | -0.5215 | No |
| 39 | PMVK |  |  | 3360 | 0.017 | -0.5381 | No |
| 40 | CDK4 |  |  | 3394 | 0.016 | -0.5436 | No |
| 41 | HDAC2 |  |  | 3407 | 0.015 | -0.5455 | No |
| 42 | RDX |  |  | 3422 | 0.015 | -0.5477 | No |
| 43 | MRPS26 |  |  | 3457 | 0.014 | -0.5534 | No |
| 44 | CBX5 |  |  | 3561 | 0.010 | -0.5709 | No |
| 45 | SNRNP25 |  |  | 3634 | 0.008 | -0.5832 | No |
| 46 | MAPRE1 |  |  | 3682 | 0.006 | -0.5912 | No |
| 47 | TMEM14C |  |  | 3705 | 0.005 | -0.5949 | No |
| 48 | H1FX |  |  | 3867 | -0.002 | -0.6224 | No |
| 49 | ARPC5L |  |  | 3870 | -0.002 | -0.6227 | No |
| 50 | ECI1 |  |  | 3888 | -0.003 | -0.6256 | No |
| 51 | COX5A |  |  | 4019 | -0.010 | -0.6478 | No |
| 52 | PGAM1 |  |  | 4056 | -0.013 | -0.6538 | No |
| 53 | NDUFA6 |  |  | 4074 | -0.014 | -0.6566 | No |
| 54 | C3orf14 |  |  | 4079 | -0.014 | -0.6571 | No |
| 55 | VPS29 |  |  | 4101 | -0.015 | -0.6605 | No |
| 56 | NDUFB11 |  |  | 4331 | -0.031 | -0.6994 | No |
| 57 | RALY |  |  | 4356 | -0.032 | -0.7031 | No |
| 58 | MAD2L2 |  |  | 4387 | -0.035 | -0.7079 | No |
| 59 | TFDP1 |  |  | 4389 | -0.035 | -0.7077 | No |
| 60 | VOPP1 |  |  | 4401 | -0.036 | -0.7091 | No |
| 61 | GTF3A |  |  | 4402 | -0.036 | -0.7087 | No |
| 62 | H2AFY |  |  | 4419 | -0.037 | -0.7111 | No |
| 63 | XRCC6 |  |  | 4437 | -0.039 | -0.7135 | No |
| 64 | MRPL18 |  |  | 4484 | -0.043 | -0.7209 | No |
| 65 | PPP1CA |  |  | 4485 | -0.043 | -0.7205 | No |
| 66 | SNRPF |  |  | 4498 | -0.044 | -0.7220 | No |
| 67 | ARPC1B |  |  | 4500 | -0.045 | -0.7217 | No |
| 68 | MRPS11 |  |  | 4528 | -0.046 | -0.7258 | No |
| 69 | MCMBP |  |  | 4551 | -0.048 | -0.7290 | No |
| 70 | CHAC2 |  |  | 4569 | -0.049 | -0.7314 | No |
| 71 | NUDT15 |  |  | 4571 | -0.049 | -0.7310 | No |
| 72 | CCND3 |  |  | 4578 | -0.050 | -0.7315 | No |
| 73 | HPF1 |  |  | 4582 | -0.050 | -0.7314 | No |
| 74 | RMI2 |  |  | 4623 | -0.053 | -0.7377 | No |
| 75 | GLO1 |  |  | 4640 | -0.054 | -0.7398 | No |
| 76 | NUDT5 |  |  | 4649 | -0.055 | -0.7406 | No |
| 77 | ITGB1BP1 |  |  | 4660 | -0.056 | -0.7416 | No |
| 78 | PIGX |  |  | 4688 | -0.059 | -0.7456 | No |
| 79 | CSRP2 |  |  | 4713 | -0.061 | -0.7490 | No |
| 80 | OLA1 |  |  | 4718 | -0.061 | -0.7490 | No |
| 81 | ASRGL1 |  |  | 4755 | -0.065 | -0.7545 | No |
| 82 | COPS3 |  |  | 4758 | -0.065 | -0.7541 | No |
| 83 | NRM |  |  | 4765 | -0.065 | -0.7544 | No |
| 84 | HNRNPF |  |  | 4783 | -0.066 | -0.7566 | No |
| 85 | NFIC |  |  | 4785 | -0.067 | -0.7560 | No |
| 86 | SNRPD3 |  |  | 4815 | -0.069 | -0.7602 | No |
| 87 | PSMA3 |  |  | 4817 | -0.069 | -0.7596 | No |
| 88 | XRN2 |  |  | 4823 | -0.069 | -0.7597 | No |
| 89 | PPIH |  |  | 4854 | -0.072 | -0.7640 | No |
| 90 | PIN1 |  |  | 4886 | -0.074 | -0.7685 | No |
| 91 | EIF4A3 |  |  | 4899 | -0.075 | -0.7697 | No |
| 92 | PSMG2 |  |  | 4914 | -0.076 | -0.7712 | No |
| 93 | MRPL17 |  |  | 4937 | -0.078 | -0.7741 | No |
| 94 | ACYP1 |  |  | 4975 | -0.081 | -0.7796 | No |
| 95 | NUP85 |  |  | 4982 | -0.081 | -0.7797 | No |
| 96 | RBBP4 |  |  | 4986 | -0.082 | -0.7793 | No |
| 97 | VKORC1 |  |  | 4996 | -0.082 | -0.7799 | No |
| 98 | CDK2 |  |  | 5015 | -0.084 | -0.7820 | No |
| 99 | IMMT |  |  | 5023 | -0.084 | -0.7823 | No |
| 100 | NUDC |  |  | 5032 | -0.085 | -0.7827 | No |
| 101 | DONSON |  |  | 5051 | -0.087 | -0.7848 | No |
| 102 | PARP1 |  |  | 5065 | -0.088 | -0.7860 | No |
| 103 | DCK |  |  | 5068 | -0.088 | -0.7854 | No |
| 104 | GGCT |  |  | 5073 | -0.089 | -0.7851 | No |
| 105 | LSM3 |  |  | 5075 | -0.089 | -0.7843 | No |
| 106 | TUBB6 |  |  | 5076 | -0.089 | -0.7833 | No |
| 107 | CHTF18 |  |  | 5077 | -0.089 | -0.7823 | No |
| 108 | NME1 |  |  | 5086 | -0.090 | -0.7826 | No |
| 109 | TPRKB |  |  | 5099 | -0.092 | -0.7836 | No |
| 110 | EEF1B2 |  |  | 5106 | -0.092 | -0.7836 | No |
| 111 | CARHSP1 |  |  | 5107 | -0.092 | -0.7826 | No |
| 112 | PARP2 |  |  | 5119 | -0.093 | -0.7834 | No |
| 113 | WDHD1 |  |  | 5120 | -0.093 | -0.7824 | No |
| 114 | DSCC1 |  |  | 5133 | -0.095 | -0.7834 | No |
| 115 | SKA3 |  |  | 5136 | -0.095 | -0.7827 | No |
| 116 | PAXX |  |  | 5146 | -0.096 | -0.7831 | No |
| 117 | UNG |  |  | 5148 | -0.096 | -0.7822 | No |
| 118 | SCCPDH |  |  | 5153 | -0.097 | -0.7818 | No |
| 119 | SUPT16H |  |  | 5179 | -0.099 | -0.7850 | No |
| 120 | MNS1 |  |  | 5212 | -0.102 | -0.7893 | No |
| 121 | LY6E |  |  | 5230 | -0.104 | -0.7911 | No |
| 122 | CMSS1 |  |  | 5247 | -0.105 | -0.7926 | No |
| 123 | YEATS4 |  |  | 5250 | -0.106 | -0.7918 | No |
| 124 | POP7 |  |  | 5259 | -0.107 | -0.7919 | No |
| 125 | CENPQ |  |  | 5266 | -0.107 | -0.7918 | No |
| 126 | FANCG |  |  | 5303 | -0.110 | -0.7967 | No |
| 127 | FADS1 |  |  | 5324 | -0.111 | -0.7989 | Yes |
| 128 | UBA2 |  |  | 5328 | -0.111 | -0.7982 | Yes |
| 129 | PPIF |  |  | 5337 | -0.112 | -0.7983 | Yes |
| 130 | UBR7 |  |  | 5340 | -0.112 | -0.7974 | Yes |
| 131 | CBR3 |  |  | 5344 | -0.113 | -0.7966 | Yes |
| 132 | SRPK1 |  |  | 5360 | -0.115 | -0.7979 | Yes |
| 133 | DPM2 |  |  | 5365 | -0.115 | -0.7973 | Yes |
| 134 | PDCD5 |  |  | 5366 | -0.115 | -0.7960 | Yes |
| 135 | SAAL1 |  |  | 5368 | -0.115 | -0.7949 | Yes |
| 136 | RCC1 |  |  | 5369 | -0.115 | -0.7936 | Yes |
| 137 | RPA2 |  |  | 5372 | -0.115 | -0.7926 | Yes |
| 138 | H2AFV |  |  | 5381 | -0.117 | -0.7927 | Yes |
| 139 | C21orf58 |  |  | 5382 | -0.117 | -0.7914 | Yes |
| 140 | COA1 |  |  | 5386 | -0.117 | -0.7906 | Yes |
| 141 | WDR76 |  |  | 5388 | -0.117 | -0.7894 | Yes |
| 142 | CKLF |  |  | 5394 | -0.118 | -0.7890 | Yes |
| 143 | KLHL23 |  |  | 5397 | -0.118 | -0.7880 | Yes |
| 144 | RECQL4 |  |  | 5399 | -0.118 | -0.7868 | Yes |
| 145 | NR2C2AP |  |  | 5400 | -0.118 | -0.7855 | Yes |
| 146 | BAZ1B |  |  | 5405 | -0.119 | -0.7849 | Yes |
| 147 | EXO1 |  |  | 5410 | -0.120 | -0.7842 | Yes |
| 148 | MGME1 |  |  | 5430 | -0.122 | -0.7861 | Yes |
| 149 | EEF1E1 |  |  | 5432 | -0.122 | -0.7849 | Yes |
| 150 | RFC5 |  |  | 5434 | -0.122 | -0.7837 | Yes |
| 151 | GNG4 |  |  | 5440 | -0.123 | -0.7832 | Yes |
| 152 | SPATA33 |  |  | 5442 | -0.123 | -0.7820 | Yes |
| 153 | RFC3 |  |  | 5450 | -0.124 | -0.7818 | Yes |
| 154 | HNRNPA3 |  |  | 5455 | -0.126 | -0.7811 | Yes |
| 155 | LRR1 |  |  | 5459 | -0.126 | -0.7802 | Yes |
| 156 | PSMD14 |  |  | 5460 | -0.126 | -0.7787 | Yes |
| 157 | PFKP |  |  | 5471 | -0.128 | -0.7790 | Yes |
| 158 | RBL1 |  |  | 5472 | -0.128 | -0.7776 | Yes |
| 159 | CCT4 |  |  | 5474 | -0.128 | -0.7763 | Yes |
| 160 | TEDC1 |  |  | 5476 | -0.129 | -0.7751 | Yes |
| 161 | POLE3 |  |  | 5481 | -0.129 | -0.7743 | Yes |
| 162 | TUBB |  |  | 5482 | -0.129 | -0.7728 | Yes |
| 163 | WDR34 |  |  | 5484 | -0.130 | -0.7716 | Yes |
| 164 | TECR |  |  | 5487 | -0.130 | -0.7704 | Yes |
| 165 | RAD21 |  |  | 5496 | -0.131 | -0.7703 | Yes |
| 166 | TEDC2 |  |  | 5504 | -0.132 | -0.7701 | Yes |
| 167 | SKA1 |  |  | 5512 | -0.133 | -0.7698 | Yes |
| 168 | KPNB1 |  |  | 5514 | -0.133 | -0.7684 | Yes |
| 169 | FUCA2 |  |  | 5517 | -0.133 | -0.7673 | Yes |
| 170 | PSMG1 |  |  | 5518 | -0.133 | -0.7658 | Yes |
| 171 | NCAPH2 |  |  | 5520 | -0.133 | -0.7645 | Yes |
| 172 | PAQR4 |  |  | 5522 | -0.134 | -0.7631 | Yes |
| 173 | ABHD3 |  |  | 5523 | -0.134 | -0.7616 | Yes |
| 174 | RAD51C |  |  | 5525 | -0.134 | -0.7603 | Yes |
| 175 | GINS1 |  |  | 5542 | -0.135 | -0.7615 | Yes |
| 176 | RBBP7 |  |  | 5546 | -0.136 | -0.7605 | Yes |
| 177 | CEP57 |  |  | 5548 | -0.136 | -0.7592 | Yes |
| 178 | ACTL6A |  |  | 5550 | -0.136 | -0.7578 | Yes |
| 179 | IER3IP1 |  |  | 5551 | -0.136 | -0.7563 | Yes |
| 180 | SUZ12 |  |  | 5556 | -0.136 | -0.7555 | Yes |
| 181 | DEPDC1B |  |  | 5570 | -0.138 | -0.7561 | Yes |
| 182 | BUB3 |  |  | 5583 | -0.141 | -0.7566 | Yes |
| 183 | HMGB3 |  |  | 5584 | -0.142 | -0.7550 | Yes |
| 184 | CENPW |  |  | 5585 | -0.142 | -0.7534 | Yes |
| 185 | DCTPP1 |  |  | 5588 | -0.143 | -0.7522 | Yes |
| 186 | CHCHD3 |  |  | 5589 | -0.143 | -0.7506 | Yes |
| 187 | PSIP1 |  |  | 5590 | -0.143 | -0.7490 | Yes |
| 188 | NCAPH |  |  | 5595 | -0.144 | -0.7480 | Yes |
| 189 | CSE1L |  |  | 5597 | -0.144 | -0.7466 | Yes |
| 190 | POLA2 |  |  | 5615 | -0.146 | -0.7479 | Yes |
| 191 | CENPJ |  |  | 5625 | -0.147 | -0.7477 | Yes |
| 192 | TEX30 |  |  | 5629 | -0.148 | -0.7466 | Yes |
| 193 | GPN3 |  |  | 5630 | -0.149 | -0.7449 | Yes |
| 194 | CTPS1 |  |  | 5635 | -0.150 | -0.7439 | Yes |
| 195 | PRIM1 |  |  | 5636 | -0.150 | -0.7423 | Yes |
| 196 | HIRIP3 |  |  | 5638 | -0.151 | -0.7407 | Yes |
| 197 | MCM6 |  |  | 5644 | -0.151 | -0.7399 | Yes |
| 198 | KIFC1 |  |  | 5645 | -0.152 | -0.7382 | Yes |
| 199 | CASP8AP2 |  |  | 5646 | -0.152 | -0.7365 | Yes |
| 200 | CCDC34 |  |  | 5647 | -0.152 | -0.7348 | Yes |
| 201 | CEP152 |  |  | 5649 | -0.152 | -0.7333 | Yes |
| 202 | CCT2 |  |  | 5654 | -0.152 | -0.7322 | Yes |
| 203 | CLIC1 |  |  | 5664 | -0.154 | -0.7320 | Yes |
| 204 | CEP135 |  |  | 5669 | -0.155 | -0.7310 | Yes |
| 205 | LSM4 |  |  | 5673 | -0.155 | -0.7298 | Yes |
| 206 | PARPBP |  |  | 5682 | -0.157 | -0.7294 | Yes |
| 207 | DSN1 |  |  | 5687 | -0.158 | -0.7283 | Yes |
| 208 | TUBG1 |  |  | 5690 | -0.158 | -0.7269 | Yes |
| 209 | MCM8 |  |  | 5701 | -0.161 | -0.7268 | Yes |
| 210 | HNRNPD |  |  | 5705 | -0.161 | -0.7255 | Yes |
| 211 | HSPB11 |  |  | 5707 | -0.161 | -0.7238 | Yes |
| 212 | MSH2 |  |  | 5711 | -0.162 | -0.7225 | Yes |
| 213 | CACYBP |  |  | 5713 | -0.162 | -0.7209 | Yes |
| 214 | E2F8 |  |  | 5717 | -0.163 | -0.7196 | Yes |
| 215 | MAGOHB |  |  | 5718 | -0.164 | -0.7177 | Yes |
| 216 | COMMD4 |  |  | 5731 | -0.168 | -0.7179 | Yes |
| 217 | PRIM2 |  |  | 5736 | -0.169 | -0.7167 | Yes |
| 218 | EIF4EBP1 |  |  | 5747 | -0.171 | -0.7165 | Yes |
| 219 | SLBP |  |  | 5748 | -0.172 | -0.7146 | Yes |
| 220 | EXOSC8 |  |  | 5751 | -0.173 | -0.7130 | Yes |
| 221 | TMX1 |  |  | 5759 | -0.174 | -0.7122 | Yes |
| 222 | NFATC2IP |  |  | 5763 | -0.175 | -0.7108 | Yes |
| 223 | CISD2 |  |  | 5764 | -0.176 | -0.7088 | Yes |
| 224 | BRIP1 |  |  | 5769 | -0.177 | -0.7075 | Yes |
| 225 | BARD1 |  |  | 5779 | -0.179 | -0.7071 | Yes |
| 226 | MASTL |  |  | 5781 | -0.179 | -0.7052 | Yes |
| 227 | MCM2 |  |  | 5785 | -0.180 | -0.7037 | Yes |
| 228 | SIVA1 |  |  | 5791 | -0.181 | -0.7025 | Yes |
| 229 | TRIP13 |  |  | 5792 | -0.181 | -0.7005 | Yes |
| 230 | TCF19 |  |  | 5796 | -0.182 | -0.6990 | Yes |
| 231 | MSH6 |  |  | 5799 | -0.182 | -0.6973 | Yes |
| 232 | HAT1 |  |  | 5803 | -0.184 | -0.6957 | Yes |
| 233 | ZWILCH |  |  | 5805 | -0.184 | -0.6938 | Yes |
| 234 | SAC3D1 |  |  | 5810 | -0.186 | -0.6924 | Yes |
| 235 | MIS18A |  |  | 5816 | -0.187 | -0.6912 | Yes |
| 236 | LSM8 |  |  | 5822 | -0.188 | -0.6900 | Yes |
| 237 | CCNE2 |  |  | 5827 | -0.190 | -0.6885 | Yes |
| 238 | FDPS |  |  | 5828 | -0.190 | -0.6864 | Yes |
| 239 | PSMC3 |  |  | 5831 | -0.190 | -0.6846 | Yes |
| 240 | TIMELESS |  |  | 5832 | -0.190 | -0.6825 | Yes |
| 241 | CMC2 |  |  | 5834 | -0.191 | -0.6805 | Yes |
| 242 | PRC1 |  |  | 5835 | -0.192 | -0.6783 | Yes |
| 243 | SLF1 |  |  | 5838 | -0.192 | -0.6765 | Yes |
| 244 | PA2G4 |  |  | 5841 | -0.193 | -0.6747 | Yes |
| 245 | FAM111A |  |  | 5844 | -0.194 | -0.6729 | Yes |
| 246 | FANCD2 |  |  | 5849 | -0.195 | -0.6714 | Yes |
| 247 | RBBP8 |  |  | 5854 | -0.195 | -0.6699 | Yes |
| 248 | CDCA4 |  |  | 5861 | -0.197 | -0.6687 | Yes |
| 249 | HNRNPAB |  |  | 5862 | -0.197 | -0.6665 | Yes |
| 250 | CDCA7 |  |  | 5863 | -0.198 | -0.6643 | Yes |
| 251 | HIST1H1E |  |  | 5865 | -0.198 | -0.6622 | Yes |
| 252 | DEGS1 |  |  | 5875 | -0.200 | -0.6615 | Yes |
| 253 | CKAP2 |  |  | 5879 | -0.201 | -0.6598 | Yes |
| 254 | SAE1 |  |  | 5888 | -0.203 | -0.6589 | Yes |
| 255 | NRGN |  |  | 5889 | -0.204 | -0.6566 | Yes |
| 256 | ANP32B |  |  | 5890 | -0.204 | -0.6543 | Yes |
| 257 | DIAPH3 |  |  | 5893 | -0.205 | -0.6524 | Yes |
| 258 | C19orf48 |  |  | 5894 | -0.205 | -0.6501 | Yes |
| 259 | BTG3 |  |  | 5895 | -0.206 | -0.6477 | Yes |
| 260 | HAUS8 |  |  | 5897 | -0.206 | -0.6456 | Yes |
| 261 | BUB1B |  |  | 5901 | -0.207 | -0.6438 | Yes |
| 262 | P4HB |  |  | 5902 | -0.208 | -0.6415 | Yes |
| 263 | KDELC2 |  |  | 5903 | -0.208 | -0.6392 | Yes |
| 264 | SNRPB |  |  | 5908 | -0.209 | -0.6375 | Yes |
| 265 | BRCA2 |  |  | 5910 | -0.210 | -0.6353 | Yes |
| 266 | SNRPD1 |  |  | 5911 | -0.210 | -0.6330 | Yes |
| 267 | HACD3 |  |  | 5916 | -0.211 | -0.6313 | Yes |
| 268 | FAM111B |  |  | 5918 | -0.211 | -0.6291 | Yes |
| 269 | XRCC5 |  |  | 5923 | -0.213 | -0.6274 | Yes |
| 270 | MMS22L |  |  | 5928 | -0.215 | -0.6257 | Yes |
| 271 | ACAT2 |  |  | 5931 | -0.215 | -0.6236 | Yes |
| 272 | CENPX |  |  | 5937 | -0.217 | -0.6220 | Yes |
| 273 | RANBP1 |  |  | 5939 | -0.218 | -0.6197 | Yes |
| 274 | SMS |  |  | 5940 | -0.218 | -0.6173 | Yes |
| 275 | PSMC3IP |  |  | 5951 | -0.222 | -0.6165 | Yes |
| 276 | RACGAP1 |  |  | 5953 | -0.223 | -0.6142 | Yes |
| 277 | ALYREF |  |  | 5955 | -0.225 | -0.6118 | Yes |
| 278 | CIP2A |  |  | 5956 | -0.225 | -0.6093 | Yes |
| 279 | POLD3 |  |  | 5957 | -0.225 | -0.6068 | Yes |
| 280 | PKMYT1 |  |  | 5959 | -0.225 | -0.6044 | Yes |
| 281 | TMEM237 |  |  | 5962 | -0.226 | -0.6023 | Yes |
| 282 | NCAPD3 |  |  | 5965 | -0.227 | -0.6001 | Yes |
| 283 | DTL |  |  | 5975 | -0.229 | -0.5990 | Yes |
| 284 | IKBIP |  |  | 5976 | -0.229 | -0.5965 | Yes |
| 285 | RTKN2 |  |  | 5977 | -0.229 | -0.5939 | Yes |
| 286 | LMNB2 |  |  | 5978 | -0.229 | -0.5913 | Yes |
| 287 | TMEM106C |  |  | 5979 | -0.230 | -0.5887 | Yes |
| 288 | CD151 |  |  | 5981 | -0.230 | -0.5863 | Yes |
| 289 | MND1 |  |  | 5982 | -0.232 | -0.5837 | Yes |
| 290 | CENPV |  |  | 5984 | -0.232 | -0.5813 | Yes |
| 291 | USP1 |  |  | 5986 | -0.233 | -0.5789 | Yes |
| 292 | KIF2C |  |  | 5996 | -0.237 | -0.5778 | Yes |
| 293 | SPC24 |  |  | 6000 | -0.238 | -0.5756 | Yes |
| 294 | LIG1 |  |  | 6002 | -0.239 | -0.5731 | Yes |
| 295 | ITGB3BP |  |  | 6005 | -0.240 | -0.5707 | Yes |
| 296 | KNL1 |  |  | 6007 | -0.240 | -0.5682 | Yes |
| 297 | POC1A |  |  | 6010 | -0.241 | -0.5659 | Yes |
| 298 | RPA3 |  |  | 6014 | -0.244 | -0.5636 | Yes |
| 299 | SMC1A |  |  | 6017 | -0.245 | -0.5612 | Yes |
| 300 | CHEK1 |  |  | 6025 | -0.248 | -0.5596 | Yes |
| 301 | CTNNAL1 |  |  | 6027 | -0.249 | -0.5570 | Yes |
| 302 | DEPDC1 |  |  | 6030 | -0.251 | -0.5545 | Yes |
| 303 | EMP2 |  |  | 6032 | -0.254 | -0.5519 | Yes |
| 304 | VRK1 |  |  | 6034 | -0.256 | -0.5492 | Yes |
| 305 | FEN1 |  |  | 6035 | -0.256 | -0.5463 | Yes |
| 306 | DNAJC9 |  |  | 6037 | -0.260 | -0.5435 | Yes |
| 307 | KDELR2 |  |  | 6038 | -0.262 | -0.5406 | Yes |
| 308 | RAD18 |  |  | 6039 | -0.263 | -0.5377 | Yes |
| 309 | SMC3 |  |  | 6041 | -0.265 | -0.5349 | Yes |
| 310 | MXD3 |  |  | 6042 | -0.265 | -0.5319 | Yes |
| 311 | GINS4 |  |  | 6048 | -0.267 | -0.5297 | Yes |
| 312 | CALU |  |  | 6049 | -0.268 | -0.5267 | Yes |
| 313 | PGRMC1 |  |  | 6051 | -0.270 | -0.5239 | Yes |
| 314 | TUBA1C |  |  | 6052 | -0.270 | -0.5209 | Yes |
| 315 | HJURP |  |  | 6055 | -0.271 | -0.5182 | Yes |
| 316 | CDC45 |  |  | 6063 | -0.276 | -0.5163 | Yes |
| 317 | ARHGAP11A |  |  | 6067 | -0.277 | -0.5137 | Yes |
| 318 | MCM5 |  |  | 6069 | -0.278 | -0.5107 | Yes |
| 319 | FOXM1 |  |  | 6070 | -0.279 | -0.5076 | Yes |
| 320 | SHCBP1 |  |  | 6071 | -0.279 | -0.5045 | Yes |
| 321 | SPC25 |  |  | 6072 | -0.279 | -0.5013 | Yes |
| 322 | GMNN |  |  | 6080 | -0.285 | -0.4993 | Yes |
| 323 | PLP2 |  |  | 6082 | -0.286 | -0.4963 | Yes |
| 324 | FBXO5 |  |  | 6084 | -0.287 | -0.4933 | Yes |
| 325 | KIF22 |  |  | 6089 | -0.290 | -0.4907 | Yes |
| 326 | CENPN |  |  | 6090 | -0.291 | -0.4874 | Yes |
| 327 | DEK |  |  | 6092 | -0.292 | -0.4843 | Yes |
| 328 | NUF2 |  |  | 6093 | -0.293 | -0.4810 | Yes |
| 329 | NCAPG2 |  |  | 6094 | -0.294 | -0.4778 | Yes |
| 330 | RFC2 |  |  | 6095 | -0.294 | -0.4745 | Yes |
| 331 | FANCA |  |  | 6097 | -0.295 | -0.4713 | Yes |
| 332 | CDT1 |  |  | 6098 | -0.295 | -0.4680 | Yes |
| 333 | CDCA5 |  |  | 6099 | -0.296 | -0.4647 | Yes |
| 334 | CCNA2 |  |  | 6100 | -0.297 | -0.4614 | Yes |
| 335 | CDC6 |  |  | 6103 | -0.299 | -0.4584 | Yes |
| 336 | DUT |  |  | 6107 | -0.303 | -0.4555 | Yes |
| 337 | CKAP2L |  |  | 6108 | -0.303 | -0.4521 | Yes |
| 338 | MCM10 |  |  | 6110 | -0.305 | -0.4488 | Yes |
| 339 | SLC20A1 |  |  | 6112 | -0.306 | -0.4456 | Yes |
| 340 | MCM3 |  |  | 6113 | -0.307 | -0.4421 | Yes |
| 341 | PIMREG |  |  | 6115 | -0.310 | -0.4388 | Yes |
| 342 | CCT5 |  |  | 6116 | -0.312 | -0.4353 | Yes |
| 343 | MELK |  |  | 6117 | -0.312 | -0.4318 | Yes |
| 344 | MIS18BP1 |  |  | 6122 | -0.316 | -0.4290 | Yes |
| 345 | PHGDH |  |  | 6126 | -0.318 | -0.4259 | Yes |
| 346 | ASF1B |  |  | 6127 | -0.320 | -0.4223 | Yes |
| 347 | PHF19 |  |  | 6129 | -0.322 | -0.4189 | Yes |
| 348 | TMEM97 |  |  | 6131 | -0.324 | -0.4154 | Yes |
| 349 | HMGA1 |  |  | 6132 | -0.324 | -0.4118 | Yes |
| 350 | LMNB1 |  |  | 6134 | -0.325 | -0.4083 | Yes |
| 351 | CDCA2 |  |  | 6135 | -0.326 | -0.4047 | Yes |
| 352 | CENPH |  |  | 6136 | -0.327 | -0.4010 | Yes |
| 353 | DDX39A |  |  | 6137 | -0.328 | -0.3973 | Yes |
| 354 | TACC3 |  |  | 6138 | -0.328 | -0.3937 | Yes |
| 355 | NDC80 |  |  | 6142 | -0.330 | -0.3905 | Yes |
| 356 | AURKB |  |  | 6143 | -0.332 | -0.3867 | Yes |
| 357 | TK1 |  |  | 6144 | -0.334 | -0.3830 | Yes |
| 358 | NUDT1 |  |  | 6146 | -0.338 | -0.3794 | Yes |
| 359 | RRM1 |  |  | 6147 | -0.338 | -0.3756 | Yes |
| 360 | PRKDC |  |  | 6149 | -0.341 | -0.3719 | Yes |
| 361 | E2F1 |  |  | 6152 | -0.343 | -0.3684 | Yes |
| 362 | KIF15 |  |  | 6158 | -0.346 | -0.3654 | Yes |
| 363 | EZH2 |  |  | 6159 | -0.348 | -0.3615 | Yes |
| 364 | CDKN3 |  |  | 6160 | -0.349 | -0.3576 | Yes |
| 365 | ATAD5 |  |  | 6161 | -0.350 | -0.3537 | Yes |
| 366 | KIF23 |  |  | 6162 | -0.351 | -0.3497 | Yes |
| 367 | KIF4A |  |  | 6164 | -0.355 | -0.3459 | Yes |
| 368 | CDCA3 |  |  | 6165 | -0.355 | -0.3419 | Yes |
| 369 | RAD51AP1 |  |  | 6167 | -0.357 | -0.3381 | Yes |
| 370 | ANP32E |  |  | 6168 | -0.357 | -0.3341 | Yes |
| 371 | CENPK |  |  | 6169 | -0.363 | -0.3300 | Yes |
| 372 | MCM4 |  |  | 6171 | -0.372 | -0.3260 | Yes |
| 373 | PCNA |  |  | 6173 | -0.374 | -0.3220 | Yes |
| 374 | DTYMK |  |  | 6177 | -0.381 | -0.3183 | Yes |
| 375 | CENPM |  |  | 6179 | -0.386 | -0.3141 | Yes |
| 376 | DBF4 |  |  | 6180 | -0.387 | -0.3098 | Yes |
| 377 | DNMT1 |  |  | 6182 | -0.396 | -0.3055 | Yes |
| 378 | CHAF1A |  |  | 6183 | -0.396 | -0.3011 | Yes |
| 379 | RFC4 |  |  | 6185 | -0.399 | -0.2967 | Yes |
| 380 | SGO1 |  |  | 6188 | -0.404 | -0.2926 | Yes |
| 381 | MYBL2 |  |  | 6190 | -0.408 | -0.2882 | Yes |
| 382 | SGO2 |  |  | 6192 | -0.413 | -0.2837 | Yes |
| 383 | GINS2 |  |  | 6194 | -0.420 | -0.2792 | Yes |
| 384 | ZWINT |  |  | 6195 | -0.425 | -0.2744 | Yes |
| 385 | TUBB4B |  |  | 6196 | -0.431 | -0.2696 | Yes |
| 386 | BRCA1 |  |  | 6197 | -0.435 | -0.2647 | Yes |
| 387 | UHRF1 |  |  | 6198 | -0.436 | -0.2598 | Yes |
| 388 | RRM2 |  |  | 6199 | -0.438 | -0.2549 | Yes |
| 389 | DHFR |  |  | 6200 | -0.441 | -0.2499 | Yes |
| 390 | ESCO2 |  |  | 6206 | -0.463 | -0.2456 | Yes |
| 391 | PBK |  |  | 6207 | -0.465 | -0.2404 | Yes |
| 392 | FANCI |  |  | 6209 | -0.469 | -0.2353 | Yes |
| 393 | XRCC2 |  |  | 6212 | -0.482 | -0.2303 | Yes |
| 394 | BIRC5 |  |  | 6214 | -0.484 | -0.2250 | Yes |
| 395 | GTSE1 |  |  | 6217 | -0.490 | -0.2199 | Yes |
| 396 | CLSPN |  |  | 6218 | -0.490 | -0.2144 | Yes |
| 397 | SMC4 |  |  | 6219 | -0.490 | -0.2089 | Yes |
| 398 | HELLS |  |  | 6221 | -0.501 | -0.2034 | Yes |
| 399 | TYMS |  |  | 6222 | -0.501 | -0.1978 | Yes |
| 400 | NUSAP1 |  |  | 6223 | -0.507 | -0.1921 | Yes |
| 401 | NCAPG |  |  | 6227 | -0.513 | -0.1869 | Yes |
| 402 | SMC2 |  |  | 6229 | -0.518 | -0.1812 | Yes |
| 403 | GGH |  |  | 6232 | -0.522 | -0.1757 | Yes |
| 404 | TMPO |  |  | 6233 | -0.522 | -0.1699 | Yes |
| 405 | CKS1B |  |  | 6234 | -0.523 | -0.1640 | Yes |
| 406 | KPNA2 |  |  | 6236 | -0.543 | -0.1581 | Yes |
| 407 | H2AFX |  |  | 6237 | -0.576 | -0.1516 | Yes |
| 408 | ORC6 |  |  | 6238 | -0.585 | -0.1451 | Yes |
| 409 | CKS2 |  |  | 6239 | -0.585 | -0.1385 | Yes |
| 410 | UBE2T |  |  | 6240 | -0.593 | -0.1319 | Yes |
| 411 | HIST1H1D |  |  | 6241 | -0.601 | -0.1251 | Yes |
| 412 | ATAD2 |  |  | 6243 | -0.607 | -0.1185 | Yes |
| 413 | CDK1 |  |  | 6246 | -0.627 | -0.1118 | Yes |
| 414 | MAD2L1 |  |  | 6247 | -0.628 | -0.1048 | Yes |
| 415 | CENPU |  |  | 6248 | -0.633 | -0.0977 | Yes |
| 416 | H2AFZ |  |  | 6250 | -0.651 | -0.0905 | Yes |
| 417 | MCM7 |  |  | 6252 | -0.665 | -0.0832 | Yes |
| 418 | TPX2 |  |  | 6253 | -0.667 | -0.0758 | Yes |
| 419 | ASPM |  |  | 6254 | -0.680 | -0.0681 | Yes |
| 420 | UBE2C |  |  | 6255 | -0.750 | -0.0597 | Yes |
| 421 | HMGB2 |  |  | 6258 | -0.766 | -0.0515 | Yes |
| 422 | PCLAF |  |  | 6259 | -0.790 | -0.0426 | Yes |
| 423 | TOP2A |  |  | 6260 | -0.814 | -0.0335 | Yes |
| 424 | MKI67 |  |  | 6261 | -0.882 | -0.0236 | Yes |
| 425 | HIST1H4C |  |  | 6262 | -1.038 | -0.0120 | Yes |
| 426 | CENPF |  |  | 6263 | -1.068 | 0.0000 | Yes |
Table: GSEA details [plain text format]

  

Fig 2: DOROSZKO\_U3180MG\_CLUSTER\_3: Random ES distribution      
 Gene set null distribution of ES for **DOROSZKO\_U3180MG\_CLUSTER\_3**
